# Effects of Endodontic Irrigants on Material and Surface Properties of Biocompatible Thermoplastics

**DOI:** 10.3390/dj7010026

**Published:** 2019-03-06

**Authors:** Michael Kucher, Martin Dannemann, Niels Modler, Christian Hannig, Marie-Theres Weber

**Affiliations:** 1 Institute of Lightweight Engineering and Polymer Technology (ILK), Technische Universität Dresden, Holbeinstraße 3, 01307 Dresden, Germany; michael.kucher@tu-dresden.de (M.K.); niels.modler@tu-dresden.de (N.M.); 2 Clinic of Operative and Pediatric Dentistry, Medical Faculty Carl Gustav Carus, Technische Universität Dresden, Fetscherstraße 74, 01307 Dresden, Germany; christian.hannig@uniklinikum-dresden.de (C.H.); marie-theres.weber@uniklinikum-dresden.de (M.-T.W.)

**Keywords:** biocompatible polymer, root canal treatment, irrigant activation, passive sonic irrigation, microindentation, hardness, elastic modulus, surface roughness, polyether ether ketone, polyamide

## Abstract

Passive irrigation is an efficient method for a successful endodontic treatment. During sonic activation biocompatible polymer tips are used to activate irrigants. Compared to ultrasonic activation with metallic tips, polymer tips have the advantage of a reduced risk of fracture and minimise dentine damage. Hence, two polymers, polyether ether ketones (PEEK) and polyamide (PA6), were identified for the manufacturing of novel irrigation tips. The chemical resistance against the irrigants ethylenediaminetetraacetic acid (EDTA) 20%, chlorhexidine gluconate (CHX) 2% and sodium hypochlorite (NaOCl) 5.25% was analysed. Using microindentation, the change of hardness, elasticity, surface roughness and appearance of the polymers was determined. PA6 had a high absorption of irrigant compared to PEEK. PEEK was resistant to the investigated irrigants and showed no significant alteration of surface and mechanical properties, whereas PA6 slightly increased its hardness, elastic modulus and surface roughness during long-term exposure at 37 °C. However, PA6 tips seem to be a promising disposable product due to the material’s high deformability and low manufacturing costs. Particularly with regard to structural-dynamic properties and high chemical resistance, PEEK can be considered as a material for reusable irrigation tips.

## 1. Introduction

Due to the complex root canal anatomy, effective irrigation with chemical agents alongside mechanical preparation is a key factor for an effective endodontic treatment. Thus, the removal of infected dentine, the smear layer, remaining pulp tissue, bacteria and their endotoxins as well as decomposition products is crucial for the endodontic success [1,2,3,4]. In order to improve the disinfection abilities of irrigants, the use of passive ultrasonic irrigation (PUI) and passive sonic irrigation (PSI) is a generally accepted method to achieve significantly better results than merely needle and syringe irrigation [5,6,7]. Sodium hypochlorite (NaOCl) serves as the main irrigating solution and is used in different concentrations [8,9]. However, NaOCl solution with a concentration of 5.25% is the most effective concentration and thus considered as gold standard [10,11]. NaOCl has tissue dissolving properties and is capable of inhibiting most microbes, spores as well as viruses [8,12]. Nevertheless, NaOCl is also known for its cytotoxicity and inability to remove the inorganic part of the smear layer [13]. In contrast to NaOCl, ethylenediaminetetraacetic acid (EDTA) has chelating abilities, is able to bind calcium and magnesium in order to decompose inorganic components of the smear layer [14]. However, EDTA has very little antibacterial effect, even when used in a concentration of 20% [15]. Another solution is chlorhexidine gluconate (CHX) which still belongs to the important endodontic irrigants with its antibacterial effects, substantively to dentine up to 72 h and low toxicity [16,17]. Especially in persistent infections and revisions of root canal fillings it is used as an additional irrigant since it has no tissue dissolving abilities and cannot remove the smear layer [18]. By means of powerful acoustic microstreaming and cavitation effects, organic tissue, planktonic bacteria and dentine debris are removed from root canals [19]. In complex curved root canals as illustrated in References [20,21], the risk is increased to remove dentine during the PUI procedure due to the interaction between the oscillating tip and the canal’s walls, especially if using endodontic metallic tips [19]. Alternatively, PSI and the usage of polymer tips obtain the possibility for a reliable and non-cutting cleaning procedure [22]. Furthermore, polymer tips have a reduced risk of fracture due to its high deformability along the canal curvature during irrigation.

In dentistry, polymers are already well-established materials. Fibre-reinforced polymers, made of glass fibre and resin matrix, are used for periodontal splints, fixed partial dentures, endodontic posts, orthodontic appliances and other indirect restorations [23]. Even a polypropylene canal brush (CanalBrush; Coltene/Whaledent GmbH, Langenau, Germany) is applied as a thin root canal instrument to remove smear layer [24,25]. Other sonic irrigation tips (EndoActivator, Dentsply De Trey GMBH, Konstanz, Germany) are made of medical grade polymer with high flexibility [26,27,28]. Sonic irrigation tips (Eddy, VDW GmbH, Munich, Germany) already consist of biocompatible thermoplastic polymer, such as polyamide (PA) that are in practical use [22,29,30]. Furthermore, Gao et al. evaluated a novel root canal cleansing method using nylon fibres [31]. Modler et al. performed preliminary experimental and numerical studies of endodontic instruments made of carbon fibre-reinforced PA6 [32]. However, PA6 has a very poor resistance to acid and a low resistance to bases [33]. A more resistant biomedical polymer, established in medicine, is polyether ether ketone (PEEK) [34], which is used for different applications ranging from coatings, load-bearing implants or biosensors [34], segmental bone replacement implants [35], plate osteosynthesis [36] or for spinal implants and devices [37]. The technically advanced thermoplastic material PEEK is biocompatible, has a high chemical resistance, low damping, good thermal and mechanical properties [33]. Due to its material properties and endurance, PEEK seems to be a promising component for irrigation tips.

For the design of new irrigation tips, the influence of irrigants on the mechanical properties of PEEK needs to be investigated and compared to the established material PA. Therefore, the aim of this study is to investigate the interaction of the two polymers with different irrigants (CHX, EDTA, NaOCl) used in endodontics.

As mentioned above, the availability of irrigation tips made of biocompatible thermoplastics demonstrates the practical use for endodontic treatments. This study quantifies the alteration of polymer’s mechanical properties which occurs after a prolonged exposure to conventional endodontic irrigants. Thus, the suitability of the investigated polymers with regard to material ageing as well as degradation of elastic properties can be evaluated. By quantifying the alteration, a recommendation for the operating time in an endodontic treatment and a material selection for endodontic instruments can be derived.

## 2. Materials and Methods

### 2.1. Specimen Preparation

Commercially available plates out of two semi-crystalline thermoplastics, namely PA6 with a thickness of t = 2 mm and PEEK t = 1 mm were investigated in this study (Table 1). From these plates, specimens with dimensions of 10 mm by 10 mm were cut out, burred and cleaned in ultrasonically activated water. Identification numbers were branded on the surface of the specimens using soldering iron to prevent the removal of the sample labelling during exposure testing in irrigants. To avoid manufacturing- or machining-related anisotropy and inhomogeneities in the material, the samples were obtained from different locations of the polymer plate. Before testing, all polymer samples were dried to ensure equal initial conditions and absorption behaviour.

### 2.2. Experimental Procedures

The experiments were divided into two groups: (a) the investigation of the polymer’s absorption of irrigant and (b) the analysis of the alteration of mechanical properties (indentation hardness, indentation modulus) and surface properties (surface roughness, surface appearance) of the polymers under the influence of different irrigants. The experimental results were statistically evaluated and used to assess the absorption behaviour as well as the chemical resistance of PA6 and PEEK to endodontic irrigants. The basis of assessment of the chemical resistance to irrigants was the change of mechanical properties, the absorption and the surface alteration. Thus, the following classification was denoted:
A-Very good resistance: only slight changes in weight, dimensions and properties, no irreversible damage caused by the mediumB-Conditionally resistant: noticeable changes in properties, irreversible damage on prolonged exposureC-Not resistant: within short time strong attack and/or stress cracking, irreversible damage

#### 2.2.1. Determination of Absorption

For the determination of absorption, the mass of each dried sample in initial state was measured by means of an analytical balance (ABT 220-5DM, Kern & Sohn GmbH, Balingen, Germany) with a precision of 0.1 mg. The averaged sample weight was m = 2784 ± 330 mg for PA6 and m = 1972 ± 169 mg for PEEK. After every storage period, a number of three samples per polymer and irrigant were excluded, dabbed and the saturated-surface-dry mass after a constant waiting period determined. The specific absorption of quality is defined as

(1) $$W_{m}=\frac{m_{b}-m_{g}}{m_{g}}100\%,$$

where $m_{b}$ is the mass of the specimen including the absorbed solution and $m_{g}$ is the mass of the dry specimen. To identify possible reaction products, the individual solutions were photographically documented with regard to discolorations or other changes in the initial state and after the end of the absorption investigations.

#### 2.2.2. Analysis of Mechanical Properties of Polymers

**Figure 1:**
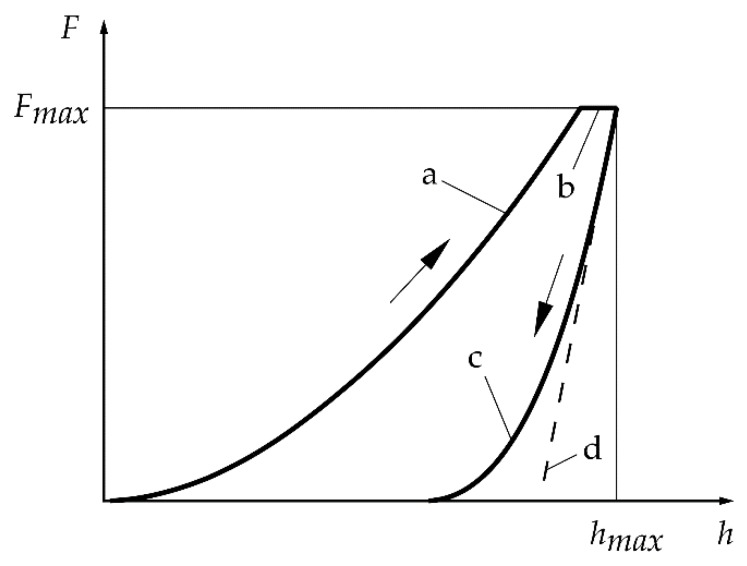
Exemplary load-displacement curve of an instrumented microindentation test: application of test force, **a**; creep at test force, **b**; decrease of test force, **c**; tangent to curve **c** at $F_{max}$, **d**.

The mechanical properties of the polymers under the influence of irrigants were analysed using instrumented indentation tests following VDI/VDE 2616 [41]. By means of an automated microindentation measuring system with programmable XY-table (Fischerscope H100C, Helmut Fischer GmbH, Sindelfingen, Germany), the hardness and elasticity of the samples were measured in a semi-automatic measurement procedure. For each test condition and the control groups, the indentation hardness using an ideal Vickers pyramid [41]

(2) $$H_{IT}=\frac{F}{24.43h_{c}^{2}}$$

was measured, where F is the test force and $h_{c}$ is the contact depth. This hardness value represents the plastic deformation of the polymer. The measurements were performed with a test force $F_{max}$ = 0.4 N, an application time of the test force $t_{F}$ = 40 s, a dwell time of the test force $t_{d}$ = 60 s and a time for removing the test force of $t_{r}$ = 40 s. The test force was applied with a constant gradient of ∂F/∂t = 0.01 N/s. During the measurement campaign, a total displacement of the indenter $h_{max}$ from 9.7 µm to 12.6 µm was determined for PA6 and from 8.8 µm to 9.5 µm for PEEK. Using the tangent of decrease curve (compare Figure 1), the indentation modulus

(3) $$E_{IT}=\frac{1-\nu_{s}^{2}}{\frac{1}{E_{r,n}}-\frac{1-\nu_{i}^{2}}{E_{i}}}$$

was measured, where $\nu_{s}$ is the Poisson’s ratio of the specimen, $\nu_{i}$ is the Poisson’s ratio of the indenter, $E_{r,n}$ is reduced modulus at contact and $E_{i}$ is the elastic modulus of the indenter. The value $\nu_{s}$ according to Table 1 was used to calculate the indentation modulus in this study. The tangent was determined by least squares approximation of the unloading curve from 65% to 95% of $F_{max}$. For each sample, a number of at least five measurements at different positions without overlapping were carried out. The determination of $H_{IT}$ and $E_{IT}$ as well as the statistical evaluation was performed using the manufacturer’s original software WIN-HCU 7.7. Curves that deviated from the curves illustrated in Figure 1 were excluded and not used for the statistic evaluation.

#### 2.2.3. Determination of Surface Roughness

The arithmetic average roughness Ra was measured by means of a three-dimensional measurement system (µscan, NanoFocus AG, Oberhausen, Germany) following DIN EN ISO 4287 [42]. The middle sections of dried specimens were investigated. For each sample, an area of 5 mm by 5 mm containing 100 lines in one direction with a spatial resolution of 5 µm in the other direction was analysed and the average of all lines was used to quantify the polymers surface roughness. Afterwards, reflected light microscopic images of selected polymer surfaces were taken and analysed. For all samples, a magnification of M = 100 was used.

### 2.3. Test Protocol

For the investigation of absorption behaviour (a), the plate-shaped samples were stored in three different irrigants with a certain mass concentration $\gamma_{i}=m_{i}/V$ (CHX, $\gamma_{i}$ = 2%; EDTA, $\gamma_{i}$ = 20%; NaOCl, $\gamma_{i}$ = 5.25%) and additionally in deionised water at a constant temperature of T = 20 °C. Glass beakers ($V_{b}$ = 600 mL) were filled with an irrigant volume of 100 mL. After the expiration of each storage time (1 h, 2 h, 6.5 h, 24 h, 48 h, 96 h, 264 h, 432 h), three samples were removed and their saturated-surface-dry mass was determined. These time increments were chosen to enable the analysis of short-term as well as long-term absorption behaviour.

**Figure 2:**
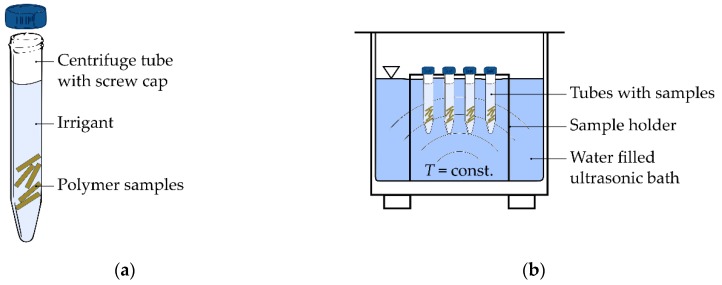
Storage of polymer samples in irrigant with a mass concentration $\gamma_{i}$ and constant temperature T: (**a**) set of samples for one chemical exposure condition and time; (**b**) arrangement of tubes for ultrasonic excitation.

The quantification of the polymer’s alteration of mechanical and surface properties during storage in irrigants (b) was investigated at temperatures of T = 20 °C (room temperature of the air-conditioned laboratory) and T = 37 °C (body temperature inside the incubator). The samples stored at T = 37 °C were additionally ultrasonically excited in an ultrasonic bath filled with deionised water (Figure 2). The same irrigants and concentrations as for the first experimental procedure were used. A set of five samples of the identical polymer was kept in a closed centrifuge tube. For each test configuration of the implemented test protocol (period of exposure, temperature, excitation), one tube was used, which resulted in a total number of 36 tubes. Five samples of each polymer were not stored and served as control groups. Every 24 h, samples were exposed to the following excitation sequence: 10 min of excitation followed by 10 min break, repeated three times. In the following, the time t = 1 d = 24 h represents one day, according to the International System of Units (SI). After the respective period of exposure (4 h, 7 d, 14 d), the samples were rinsed with water and afterwards dried for at least 12 h at a temperature of T = 80 °C in a convection oven according to manufacturer’s specifications for drying PA6. A storage time of 4 h represented a short-term application for sterile single-use instrument, whereas the periods of 7 d and 14 d simulated a repeated application. To prevent moisture absorption, especially of PA6 samples after drying, all specimens were kept in laboratory bottles with screw cap. The mechanical properties and surface roughness of the stored dried samples were measured and compared to the properties of the samples at initial state. 

## 3. Results

### 3.1. Absorption Behaviour of Polymers

**Figure 3:**
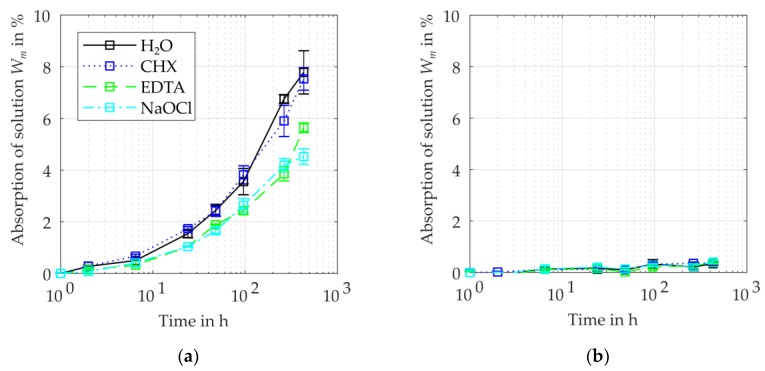
Specific absorption $W_{m}$ of irrigant and water for the investigated thermoplastic polymers: (**a**) PA6 samples; (**b**) PEEK samples.

The absorption of the different solutions after a storage time of t = 18 d varies from 4.5% to 7.8% for PA6 and from 0.3% to 0.4% for PEEK (Figure 3). In the case of PA6, $W_{m}$ obtain the highest values for water (H_2_O). For the considered time interval, no asymptotic approximation was measured, thus the equilibrium water contents will be at higher values (compare Table 1). The absorption of PEEK resulted in approximately equal values for the investigated irrigants, whereas the values for PA6 vary according to the kind of irrigant. The samples stored in CHX solution showed a similar absorption as the samples immersed in H_2_O. During the period of exposure, PA6 absorbed less EDTA and NaOCl solution than CHX solution. Water and CHX solution had the highest absorption rates, followed by EDTA and NaOCl solution.

**Figure 4:**
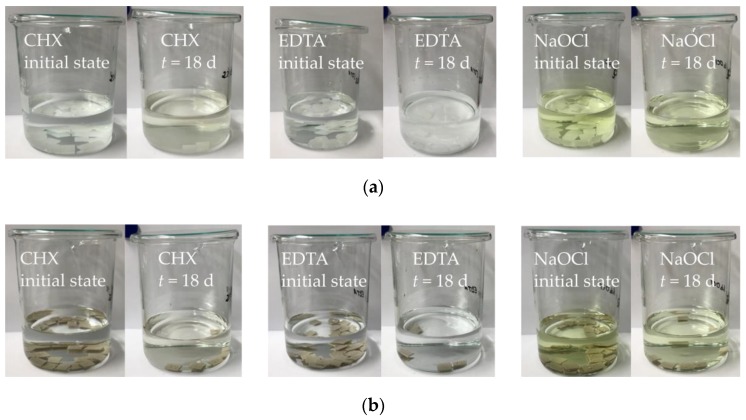
Alteration of irrigants during absorption testing: (**a**) PA6 samples; (**b**) PEEK samples.

Considering the different irrigants after polymer’s exposure, a precipitation reaction took place for EDTA solution containing PA6. A visible amount of precipitate was created and the solution turned into whitish colour (Figure 4a). For CHX and NaOCl solution containing PA6, there was no apparent change of irrigant’s colour or the forming of precipitate. The same applies to the PEEK samples immersed in the different irrigation solutions.

### 3.2. Alteration of Mechanical Properties

**Figure 5:**
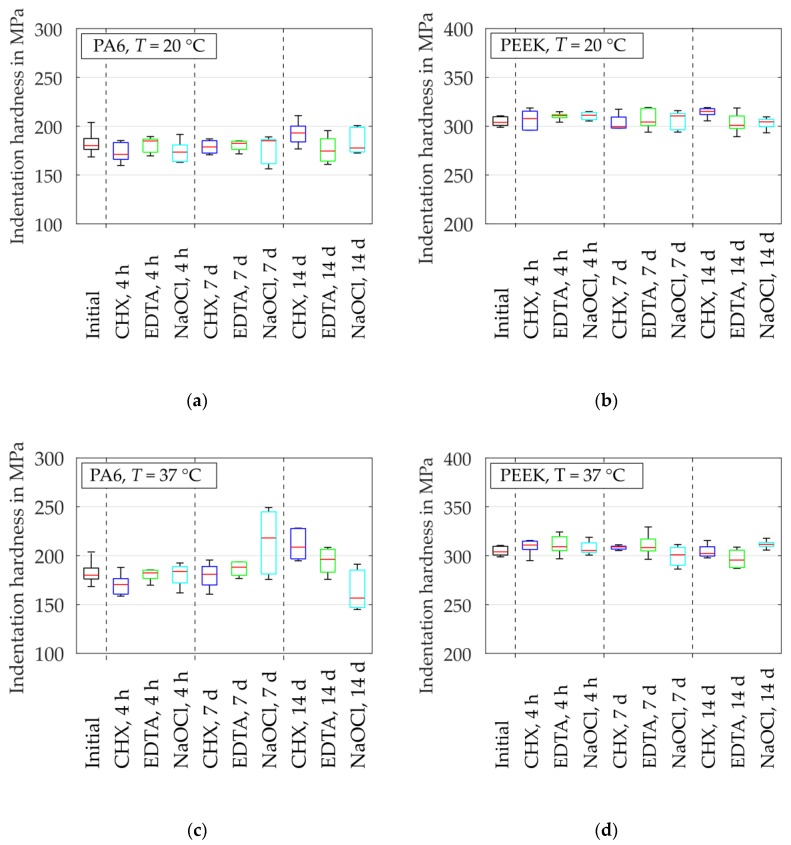
Alteration of material indentation hardness $H_{IT}$ (dried samples) after different storage times: (**a**) PA6 at T = 20 °C; (**b**) PEEK at T  = 20 °C; (**c**) PA6 at T = 37 °C and ultrasonic excitation; (**d**) PEEK at T = 37 °C and ultrasonic excitation.

At initial state, the median indentation hardness obtained a value of $H_{IT}$ = 180 GPa for PA6 and $H_{IT}$ = 304 MPa for PEEK (Figure 5). For PEEK, there was no significant change of $H_{IT}$ after exposure in irrigants at T = 20 °C as well as at T = 37 °C with additional ultrasonic excitation. However, the hardness of PA6 increased slightly after exposure in CHX, EDTA and NaOCl solution at T = 20 °C. At higher temperatures and ultrasonic excitation, this hardness increase occurred already after a shorter storage time of t = 7 d. The PA6 samples immersed in CHX and EDTA solution showed a continuous increase of hardness for both investigated temperatures after t = 7 d and t = 14 d. The hardness increase of PA6 immersed in NaOCl solution at 37 °C increased after a storage time of t = 7 d and decreased after t = 14 d. Furthermore, the standard deviation grew with increasing period of exposure for the PA6 samples.

**Figure 6:**
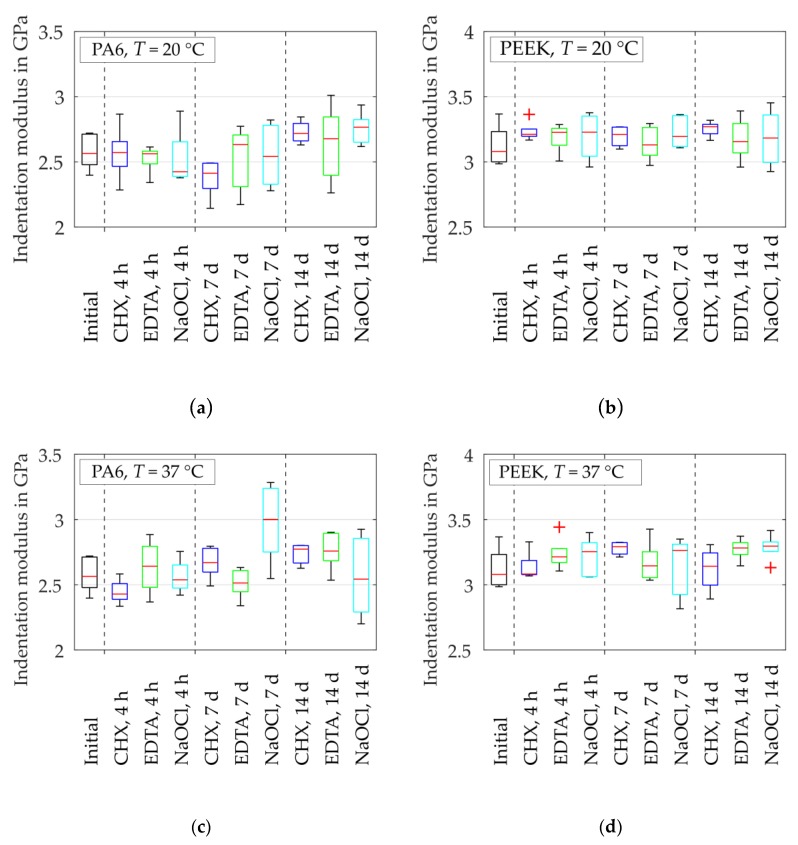
Alteration of indentation modulus $E_{IT}$ (dried samples) after different storage times: (**a**) PA6 at T = 20 °C; (**b**) PEEK at T = 20 °C; (**c**) PA6 at T = 37 °C and ultrasonic excitation; (**d**) PEEK at T  = 37 °C and ultrasonic excitation.

The median values at initial state were $E_{IT}$ = 2.57 GPa for PA6 and $E_{IT}$ = 3.08 GPa for PEEK (Figure 6). According to the manufacturer’s datasheet, the tensile modulus of elasticity is $E_{t}$ = 3 GPa for PA6 [43] and $E_{t}$ = 4 GPa for PEEK [39]. This yields the disagreement of an underestimated Young’s modulus by 0.86 times for PA6 and by 0.77 times for PEEK (compare Table 2). The indentation modulus of PA6 slightly grew with an increasing period of exposure in CHX, EDTA and NaOCl solution. For PEEK, no clear tendency was identified since the values vary around the median value of the initial state.

### 3.3. Surface Alteration

**Figure 7:**
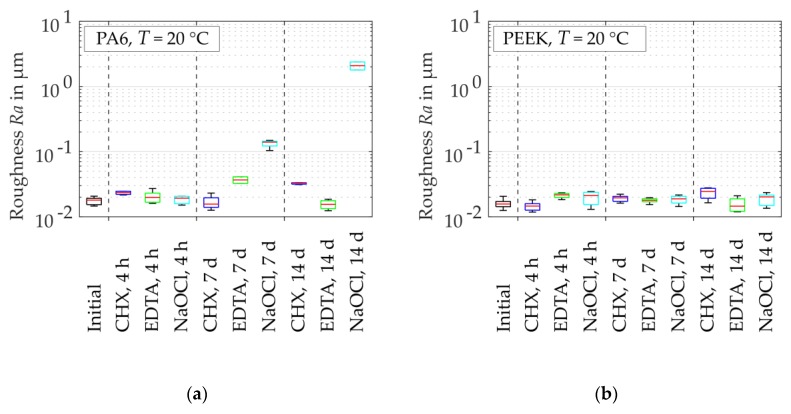

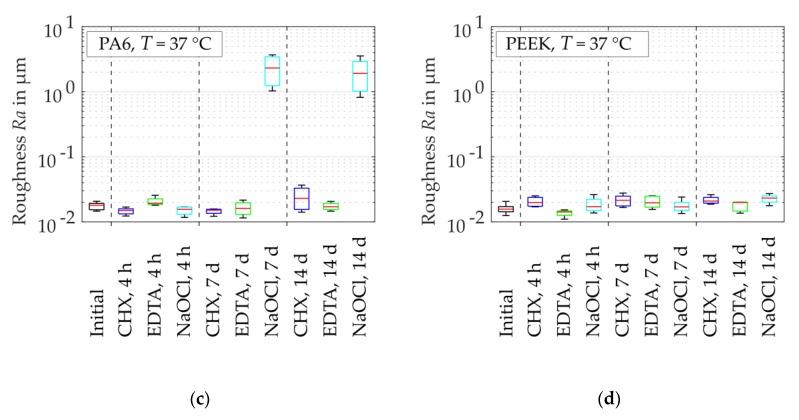
Alteration of surface roughness Ra (dried samples) due to absorption of irrigant after different storage times: (**a**) PA6 at T = 20 °C; (**b**) PEEK at T = 20 °C; (**c**) PA6 at T = 37 °C and ultrasonic excitation; (**d**) PEEK at T = 37 °C and ultrasonic excitation.

According to the manufacturer’s conditions and the sample preparation, the median surface roughness was Ra = 0.018 µm for PA6 and Ra = 0.016 µm for PEEK. The roughness of the immersed PEEK did not significantly change at T = 20 °C and T = 37 °C at the different periods of exposure (Figure 7). The roughness of PA6 stored in EDTA solution varied around the initial value. For CHX solution at T = 20 °C, the roughness increased to Ra = 0.033 µm and obtained values of Ra = 2.085 µm for NaOCl solution after a storage time of t = 14 d. Similar results were obtained for the sample stored in CHX solution and NaOCl solution at T = 37 °C with ultrasonic excitation. For these conditions, the roughness grew up to Ra = 0.023 µm and Ra = 1.91 µm, respectively. Considering the total displacement of the indenter from Section 2.2.2, a ratio from h/max(Ra) = 4.1 to h/max(Ra) = 5.3 for PA6 stored in NaOCl solution and from h/max(Ra) = 317.7 to h/max(Ra) = 343 for PEEK stored in NaOCl solution, where max(·) is the maximum value.

**Figure 8:**
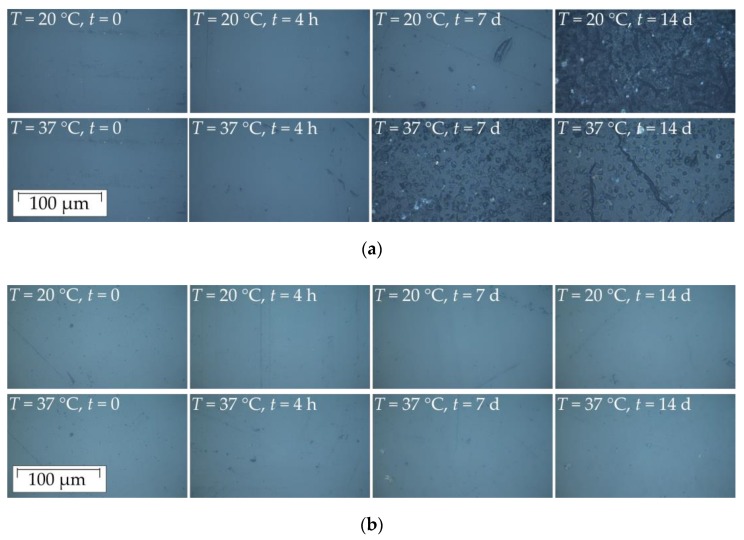
Microscopic images (magnification M = 100) illustrating the alteration of selected polymer surfaces stored in NaOCl solution at different temperatures after different storage times: (**a**) PA6 samples; (**b**) PEEK samples.

The microscopic images show for all samples manufacturing-related scratches, inclusions and imperfections, which influenced the measured surface roughness. Considering all images, only visible alterations for the PA6 samples stored in NaOCl solution were detected. The surface appearance of PA6 immersed in NaOCl solution showed no significant change after a storage time of t = 4 h. After t = 7 d at temperature of T = 37 °C and ultrasonic excitation, holes on the polymer surface were identified after drying (Figure 8a). At storage time of t = 14 d, the surface of PA6 showed cracks at both temperatures. For the PEEK sample, no holes or cracks were visible after the chemical exposure testing.

### 3.4. Chemical Resistance Chart of PA6 and PEEK

Short-term resistance was denoted for the impact on the polymers after a storage time of t = 4 h and long-term resistance after a period of t = 14 d, respectively. In general, the chemical resistance of PA6 and PEEK was not equal and both polymers showed different weight increases due to absorption of irrigant. For the investigated polymers, the classification of chemical resistance (see Section 2.2) was summarised (Table 3). Both polymers showed very good resistance (classification A) to endodontic irrigants for short-term applications, whereas PA6 was only conditionally resistant (classification B) for long-term usage.

## 4. Discussion

Passive irrigation is an efficient method to remove debris and smear layer in human root canals. Van der Sluis argued higher efficiency of PUI versus PSI due to the positive relationship between streaming velocity of the irrigant and frequency [19]. Newer studies by Neuhaus et al. showed that PSI at a frequency of f = 6000 Hz can be equal to PUI with respect to the reduction of the microbial load in curved and straight root canals [22]. Analogously, Urban et al. investigated that a sonic activation system performed equally to PUI in straight canals [29]. Nevertheless, polymer irrigation tips have the advantage to follow canal curvature, have a favourable failure behaviour and lower Young’s modulus than dentine of 20–25 GPa [45] resulting in neglectable dentine damage. However, polymers have a high material damping and, thus, self-heating effects can occur, especially for deformations at ultrasonic frequency range [46]. This effect can be reduced by using lower frequencies which results in lower streaming velocities and cavitation effects [19]. Due to the increased usage of polymer irrigation tips, the knowledge about material properties and application limitations of already existing tips as well as novel biocompatible polymers, already used in medicine, is an important field of research.

To obtain an assessment of the chemical resistance to irrigants, the absorption behaviour, the indentation hardness, the indentation modulus and the surface roughness of the established polymers PA6 [22,29] as well as the novel polymer PEEK were measured. These mechanical properties of the polymer samples were determined after certain storage times in the following irrigants: CHX 2%, EDTA 20% and NaOCl 5.25%. This approach enabled the efficient investigation of 190 specimens using small quantities of irrigants. The storage tests were performed at room temperature and body temperature to ensure practical relevance. The additional ultrasonic excitation at T = 37 °C should imitate the impact of ultrasonic irrigation treatment in endodontics. By means of a three-dimensional measurement system, the polymer’s surfaces were measured after irrigant storage and resulted especially in the alteration of surface appearance and roughness regarding PA6 immersed in NaOCl solution. In long-term applications, the chemical resistance of PA6 regarding the investigated irrigants cannot be ensured due to its high absorption of irrigants. Especially for the polymer PA6 immersed in NaOCl solution, it can be assumed that the irreversible damage will increase and that the change of mechanical properties will continue for storage times longer than t = 14 d. Between PA6 and the EDTA solution, a precipitation reaction took place. Whether these reactions result in a health risk or in a negative influence on the success of the treatment must be investigated in further studies. The absorption of the irrigant NaOCl obtained swelling, which creates shrinkage patterns, stress cracking and irreversible damage after the specimens had dried out. In literature these findings demonstrated similar results, PA6 was not resistant to NaOCl 10% at T = 20 °C after longer periods of exposure [47]. For PEEK, however, no sign of surface alteration or change of hardness as well as elastic modulus after storage was detectable. The performed microindentation measurements of PA6 and PEEK using a Vickers pyramid showed an underestimation of elastic modulus by 0.8–1 for PA6 and 0.77–0.82 for PEEK compared to manufacturer’s values. In contrast to the values of the manufacturer’s tensile testing, the indentation modulus was determined using the unloading curve after applying test force. Similarly, Tranchida et al. determined deviations regarding Young’s modulus and enumerated the sample’s varying local properties, pile-up and viscoelastic effects [48]. Furthermore, manufacturing- or machining-related effects can influence the material properties [41]. In the present study, the storage of PA6 in NaOCl solution resulted in severe surface roughness exceeding the test requirements h/Ra ≥ 20 [44] and thus the indentation modulus of PA6 has to be considered critically for these storage tests (compare Table 2). The high surface roughness causes uncertainty in the contact surface due to contact with uneven surface at small displacement of the indenter [41]. The results of the change of properties of both polymers with respect of effects of surrounding irrigants are summarised in a chemical resistance chart (Table 3).

For the design of irrigation tips, a biocompatible and enduring material is required. Already used sonic irrigation tips demonstrate the applicability of PA as sterile packed tips for disposable usage [22]. For efficient endodontic irrigation, high streaming velocity and frequency are required which are introduced by oscillations of irrigation tips [19]. The vibration patterns and therefore the resulting microstreaming and cavitation depend on the material properties of the irrigation tips. The effect of water absorption as well as the chemical exposure influences the mechanical properties. Therefore, the material properties of irrigation tips should stay constant when exposed to irrigants to ensure steady vibration behaviour. As mentioned previously, PEEK is a biocompatible polymer with high resistance to endodontic irrigants, low absorption of water and low material damping. Even in aspects regarding sterilisability, PEEK was able to endure an autoclaving process at 121 °C in water for 10 d and absorbed less than 0.4% water [49]. However, PA seems to have some deficits concerning repetitive endodontic application, such as the low resistance to chemical reagents [38], the high absorption of water [30] and the high material damping. To overcome these drawbacks, the usage of other polymers has to be taken into consideration. Hence, the polymer PEEK appears to be a novel material for endodontic applications such as oscillating irrigation tips even regarding environmental sustainability.

## 5. Conclusions

For short-term usage up to four hours, the polymers polyether ether ketone (PEEK) and polyamide (PA6) seem to be suitable materials for irrigation tips, however due to high resistance to the irrigants ethylenediaminetetraacetic acid (EDTA), chlorhexidine gluconate (CHX) and sodium hypochlorite (NaOCl), low water absorption as well as a low damping characteristic PEEK can be recommended even for long-term usage. Further investigations are required to show the applicability of polymers for irrigation tips.

## Figures and Tables

**Table 1:** Properties of dry polyamide (PA6) and polyether ether ketone (PEEK) at 20 °C.

Properties	Unit	PA6	PEEK
Modulus of elasticity (tension)	GPa	2.6…3.2 [30]	3.6…4 [38,39]
Yield stress	MPa	70…90 [30]	92…110 [38,39]
Poisson’s ratio	-	0.33 [30]	0.4 [38]
Loss factor tan δ	-	0.04 ^1^	0.01 ^2^
Melting temperature	°C	217…221 [30]	334…343 [38,39]
Service temperature	°C	−30…130 [30]	−60…250 [39]
Density	g/cm³	1.12…1.14 [30]	1.31 [39]
Water absorption	%	9…11 [30]	0.2…0.5 [38,39]

^1^ Value extracted for 20 °C from dynamic mechanical analysis performed at a frequency of f = 1 Hz [30]; ^2^ Value extracted for 20 °C from dynamic mechanical analysis performed at a frequency of f  = 10 Hz [40].

**Table 2:** Deviations between indentation modulus $E_{IT}$ (median value) and Young’s modulus measured by tensile testing $E_{t}$ from [39,43].

Duration t	Temperature T	Ultrasound Bath	$E_{IT}/E_{t}$		
			CHX	EDTA	NaOCl
$\mathrm{PA}6\;(\mathrm{initial}\;\mathrm{ratio}\;E_{IT}/E_{t}\;$= 0.856)					
4 h	20 °C	inactive	0.86	0.85	0.81
7 d			0.8	0.88	0.85
14 d			0.91	0.89	0.92 *
4 h	37 °C	active	0.81	0.88	0.85
7 d			0.89	0.84	1 *
14 d			0.92	0.92	0.85 *
$\mathrm{PEEK}\;(\mathrm{initial}\;\mathrm{ratio}\;E_{IT}/E_{t}\;$= 0.77)					
4 h	20 °C	inactive	0.83	0.81	0.81
7 d			0.80	0.78	0.80
14 d			0.82	0.79	0.80
4 h	37 °C	active	0.77	0.80	0.81
7 d			0.82	0.79	0.82
14 d			0.79	0.82	0.82

* Surface roughness exceeded materials testing requirements of surface roughness h/Ra ≥ 20 [44].

**Table 3:** Chemical resistance of polyamide (PA6) and polyether ether ketone (PEEK).

Medium	Resistance PA6 at T		Resistance PEEK at T		Weight Increase *
	Short-term	Long-term	Short-term	Long-term	
CHX, 2%	A, 20 °C A, 37 °C	B, 20 °C B, 37 °C	A, 20 °C A, 37 °C	A, 20 °C A, 37 °C	PA6: 7.5%
					PEEK: 0.4%
EDTA, 20%	A, 20 °C A, 37 °C	B, 20 °C B, 37 °C	A, 20 °C A, 37 °C	A, 20 °C A, 37 °C	PA6: 5.6%
					PEEK: 0.4%
NaOCl, 5.25%	A, 20 °C A, 37 °C	B, 20 °C B, 37 °C	A, 20 °C A, 37 °C	A, 20 °C A, 37 °C	PA6: 4.5%
					PEEK: 0.4%

* Weight increase due to absorption after storage time of t = 14 d.
